# Corporate Governance and Humble Leadership as Antecedents of Corporate Financial Performance: Monetary Incentive as a Moderator

**DOI:** 10.3389/fpsyg.2022.904076

**Published:** 2022-06-22

**Authors:** Sajjad Zahoor, Shuili Yang, Xiaoyan Ren, Syed Arslan Haider

**Affiliations:** ^1^Economics and Management School, Xi'an University of Technology, Xi'an, China; ^2^Department of Management, Sunway University, Bandar Sunway, Malaysia

**Keywords:** corporate governance, corporate financial performance, humble leadership, monetary incentive, China

## Abstract

Investors' confidence in the financial market is boosted by good corporate governance (CG). Good governance builds trust and improves an organization's financial performance (FP). However, organizations with bad management lose the trust of their stakeholders because they do not perform well financially. Therefore, the purpose of this study is to examine the influence of CG 89; on FP through mediating the role of humble leadership (HL) and monetary incentive (MI) as a moderator between CG and HL. Data were collected from 300 respondents who were working in various cement manufacturing organizations located in different cities of Pakistan. The analysis was performed using SPSS software version 25 and AMOS version 22 software to work out the study sample size. The result revealed that the framework of CG has a positive impact in terms of FP. Furthermore, HL positively and significantly mediates on CG, and FP is inextricably linked. However, MI acts as a moderator between CG and HL, but despite strengthening, it weakens the impact of CG' on HL. This study contributes toward the literature, specifically toward the expectancy theory literature. Finally, some theoretical and practical implications at the organizational level are offered, describing how CG influences FP within the organization, and research limitations and future directions are presented.

## Introduction

Corporate governance (CG) is a framework within which organizations are organized and controlled (Chowdhury et al., 2020; Fang et al., 2020). It is a “system by which the interests of the different partners are estimated” (Vintila et al., 2015). It mirrors the arrangement of connections between the organization's administration, its board, its investors, and different partners (Centobelli et al., 2019). In this research, the central point is humble leadership (HL) and the monetary incentive (MI) in CG and their effect on the financial performance (FP) of Pakistani cement manufacturing organizations (Kirkland et al., 2021). Cement manufacturing organizations assume a fundamental part in the country's financial turn of events. At the time of freedom of Pakistan in 1947, there were just four units in the country with a limit of 470,000 tons of cement production yearly, these units were situated in different cities of Pakistan, i.e., Karachi, Rohr, Wan, and Dandot (Cheema and Din, 2013). Currently, the operational capacity of cement production in Pakistan is estimated to be between 46 and 49 million tons, with an expected expansion to 76 million tons by 2020 (Salim et al., 2020). As indicated by the Pakistan Economic Survey 2016–2017, future possibilities stay productive because of advancement. In addition to the much-anticipated China Pakistan Economic Corridor- (CPEC-) related projects, there will be a slew of significant infrastructure projects, housing programs, and increased spending. Because of these variables, the use of cement in 2018–2019 is expected to reach the upper single-digit range (Nawaz et al., 2020). Therefore, in the cement industry, CG is apprehensive about addressing creations (El Gammal et al., 2020; Iqbal et al., 2020). A business thing does not mean simply being an association; however, it covers the entire business. The CG guarantees the confinement of all gatherings associated with the capture. Coordinated corporate administration adds financial solidness by improving the presentation of associations and expanding their entitlement to non-benefit pay (Shahzad et al., 2015).

For Pakistan's economic growth, CG is essential as it plays a critical role in the economic development of a country. Securities and Exchange Commission of Pakistan (SECP) regulatory actions focus on encouraging investor trust to sustain good CG and ensure responsibility of all stakeholders, especially minority interests, in Pakistan's business community. Several studies have been conducted to analyze the relationship between CG and FP in various nations, i.e., the USA (Vintila et al., 2015), Gulf Cooperation Council (GCC) (Pillai and Al-Malkawi, 2018), Indonesia (Saifi, 2019), and India (Sar, 2018). Ahmed et al. (2018) demonstrated that CG has a positive relationship with corporate performance in Pakistan. Çetinel and Güngör Tanç (2020) also examined the connection between CG and the monetary exhibition of firms. They uncovered that there is a positive yet critical connection among the construction of board structure and the board dimensions, for example, free variety and strong execution. The status of the chief executive officer (CEO) has a positive relationship and solid performance. The idea of expectation as utilized in the inspiration of laborers is frequently made by Vroom (2005). Tolman (1932) composed that live things increase expectations to understand behavioral outcomes and in this manner act in a way that may have an impact on the end outcome. Furthermore, expectancy theory recommends that the inspirational power an individual gets to pick a solitary activity from a bigger set is another capacity of the apparent potential that such conduct will bring about the accomplishment of different valence impacts of these people's results (Vroom, 2005). The emphasis of expectancy theory is on the significant phases of the arrangement of inspiration and how they are associated with and balance the impacts of specific necessities or prerequisites that may motivate behavior (Soyoung and Sungchan, 2017).

This study underpins the prescient thought that an individual will carry on or act with a specific goal in mind as the person is urged to pick a specific character over others in light of what they anticipate from the result of that specific conduct. If CG motivates leaders through MIs, the desired performance can be achieved (Zhou et al., 2021). At present, the Pakistan cement industry is developing and needs inspired laborers for further improvement (Shahzad et al., 2015). Employees are an important factor of any industry, especially labor-intensive industries such as the cement industry. Satisfied leaders can satisfy their laborers, and leaders can be satisfied through MIs. Therefore, in this study, MI is used as a moderator on the relation between CG and HL. Moreover, this research may help the management to plan and implement CG and MI schemes for executives to motivate them to achieve the desired financial results.

Therefore, the main purpose of this research is to determine whether CG and FP have a relationship through the mediating role of HL and the moderating role of MI with special emphasis on Pakistani cement manufacturing enterprises listed on the Pakistan Stock Exchange. The current study shows that decisions on CG measures should be based on wider coverage for better generalization of opinion because such decisions enhance the value of shareholders. This extension provides empirical evidence supporting the prior literature's argument that CG increases a firm's value (Chong and Lopez-de-Silanes, 2007; Fang et al., 2020).

## Literature Review

### CG and Corporate FP

Corporate governance is a blend of rules, systems, or laws that apply to, monitor, or control organizations (Chowdhury et al., 2020). The term incorporates internal and external variables that influence the interests of the organization's partners, including investors, clients, providers, government controllers, and chiefs (Çetinel and Güngör Tanç, 2020). FP decides how well an organization creates income and deals with its resources, liabilities, and monetary interests of its members. FP in organizations can be estimated through working measures. Therefore, the expectancy theory is that people make decisions among other behavioral systems based on their expectations of the extent to which a given behavior can lead to desirable outcomes (Lawler and Suttle, 1973). Some examinations look at the general effect of good governance regarding explicit approaches and best practices, while others inspect the connection between a specific part of CG and FP (Vintila et al., 2015; Mgammal et al., 2017). This study will momentarily discuss both of these subjects in this segment. According to Pillai and Al-Malkawi (2018), there is a significant amount of adolescence with regard to CG rehearses as a rule in the GCC. An incredible factor is the nonappearance of legal specialists, which makes it seem less official to follow CG codes in GCC nations and to keep CG content following norms.

Likewise, investigation by Chong and Lopez-de-Silanes (2007) showed that how huge administration rehearses have expanded corporate accounts and high productivity. The outcomes suggest that better worth creation is connected to firms with more excellent exposure, a higher spotlight on outer proprietorship, and a spotlight rather on the turn of events. Moreover, research conducted in the UK inspected the effect of CG on organizational activities using an example of 435 openly recorded monetary firms during the time frame 1999–2009 (Akbar et al., 2020). They have established using a governance index that will assist them to decide the effect of CG and performance. They see great governance as an approach to adjust the interests of different investors and partners, including providers, clients, chiefs, and supporters. In light of the above discussion, we propose the following hypothesis:

*H1: CG has a favorable relationship with corporate FP*.

### CG and HL

Corporate governance implies a bunch of rules, practices, and strategies that an organization is represented by El Gammal et al. (2020). The reason for CG is firmly connected to the meaning of a word reference. The standards of governance express that the organization's targets should be met as per the standards of the association and should be successfully executed, overlooked, moved, or changed to support individuals (Mohamad, 2018). CG is composed of various components, including that the organizational board of the association ought to have profoundly qualified chiefs with key characters and broad business knowledge (Owens et al., 2013). The expectancy theory framework argues that to make efforts one must have a clear expectation that increased effort will be associated with results considered valent (Vroom, 2005). The governing body supervises and coordinates the exercises of the chiefs of the top organizations, as they are the leaders working in the association. Accordingly, a solid connection between the top managerial staff and the board is basic to acceptable governance. The role of directors and chiefs is a vital piece of CG. The feasibility of the association's governance system, the attitude, and the mindset of chiefs who elected to serve on the board were all taken into consideration (El Gammal et al., 2020).

Another significant factor in CG is the board setup. It implies how an association isolates its administrators and shows the significance of the roles of leaders in CG. In this change, senior heads of the organizations play out their obligations of directing all organizational tasks and guarantee that they are responsible for all choices made by the organization. The study refers to suggestions that associations need suspensions made in zones with fewer chiefs because numerous degrees of executives will generally postpone dynamic and auditing measures and may hide any criminal behavior (Chowdhury et al., 2020). Viable CG coordinates the uprightness of the association, and such trustworthiness straightforwardly affects individual honesty from top administration to extremely low-level workers. Seijts et al. (2019) clarify that organization heads take a gander at or should take a gander at three things when recruiting, reviewing, and, once in a while, firing the manager: abilities, responsibility, and morals. These are some issues that are pertinent to CG. Employees' competency in an association implies having the option to achieve an assignment. Responsibility implies giving of oneself to something or an affair. It decides how well representatives are ready for the penances necessary to accomplish the objectives of the association (Sohail et al., 2020). The idea of human resource (HR) clarifies how individuals utilize their abilities, which are perceived inside and outside the association. An authoritative setting directs how a choice is settled on and how these choices are made and assessed in the association. Abilities, responsibility, and initiative character, to a significant extent, make use of the inspiration and dependability of the association. A successful CG is an incredible propelling component that straightforwardly affects the representatives to build their proficiency and profitability; subsequently, the association turns out to be more beneficial and accomplishes the objectives of the association. Therefore, based on the reviewed literature, Hypothesis 2 has been developed:

*H2: There is a positive association among CG and HL*.

### HL and Corporate FP

Humility is characterized as a personal attribute that emerges in social circumstances and demonstrates a readiness to assess oneself properly, an appreciation for the talents and contributions of others, and a willingness to learn (Kirkland et al., 2021). Humble people are also known to own their flaws in addition to their talents, seek diverse input, and value the contributions of others. In a similar spirit, Nielsen et al. (2010) defined humility as “a desirable personal attribute that express the desire to comprehend the self (identities, strengths, and limits), paired with a perspective in the self's connections with others.” From this, we may conclude that leadership is a dyadic social interaction between leaders and followers (Wen et al., 2019), i.e., leaders and followers engage with one another (as noted by Chowdhury et al., 2020). A modest leadership style is characterized by its bottom-up orientation and its reliance on three primary practices: listening, watching others, and learning by action. In fact, according to a recent study, humble leaders provide an extra humanistic approach that includes openness, listening to what followers think of them, a nice demeanor, admitting to requiring advice, and assisting followers—therefore eliminating a power gap. In general, the modest leadership behavior promotes workers to produce and share new ideas in organizations, and humble leaders are more likely to embrace new ideas and learn from others as they are conscious of their limits.

There are various sorts of pioneers who maintain a business and the degree of responsibility and accountability decides their jobs and the significance of adding to the monetary targets of the business (Kirkland et al., 2021). In CG, it is important to adjust the senior administration to the organization's advantages, thusly, the association will accomplish its drawn-out monetary objectives (Çetinel and Güngör Tanç, 2020). The achievement of any association relies on the strength of its individuals. Are the individuals ready to do what the association does? The nature of CG, authoritative governance, is a significant issue that is examined in the corporate area. They identify each other. The researcher clarified that governance is the capacity to impact a gathering in accomplishing an aim or set of objectives (Wen et al., 2019). Leaders and executives are regularly confounded, although they are important for the association.

Wajdi (2017) states that initiative and executives are two discrete and intelligent frameworks of activity. Each has his obligations and duties. Leaders are linked to the management of difficulty. Authority, by correlation, is opposed to adaptation. Numerous organizations have neglected to increment and guarantee authoritative profitability, bringing about great governance, even though they had talented staff and the correct workplace (Ran et al., 2021). Business leaders in these organizations have not had the option to spur their kin by actualizing significant worker motivation (Kirkland et al., 2021). Staff assets should be convinced to urge them to improve in the association. With this, organizational leaders need to propel themselves first to persuade others. On the off chance that they do not feel that they are planning something to improve the individuals and their associations, aside from their typical exercises, it may not be difficult to manage the difficulties and changes of existing and future organizations. Associations need solid CG and executives to work viably and meet corporate objectives (Bonet and Salvador, 2017; Chowdhury et al., 2020). As a result, we can formulate a hypothesis:

*H3: HL is positively associated with corporate FP*.

### HL as a Mediator

As indicated in past research, CG and FP are acceptable connections, but HL can influence their relations. HL has created a wide scope of interest in scholastic and innovative networks throughout the long term (Samanta and Lamprakis, 2018; Kirkland et al., 2021). HL is the way toward getting individuals to give a valiant effort to accomplish the objective you need and can be characterized as the ability to convince others to carry on unexpectedly (Armstrong et al., 2018; Zeb et al., 2018; Naidoo, 2019).

Abdalla (2015) proposes that HL is a cycle of impact on the exercises of a coordinated gathering to in its endeavors accomplish certain objectives. The principal techniques for authority see this idea as “a three-dimensional, inside, and autonomous cycle in which only the character, attributes, or characteristics of a leader are thought of” (Chow et al., 2017). Thereafter, scientists added status components to the idea of governance by underscoring that a leader should change his/her incentive style in the workplace and particularly in the degree of development of subordinates, sort of exercises (Pawirosumarto et al., 2017). There is a significant job for an initiative in accomplishing best practices and FP. Other significant rules of CG can incorporate board size, autonomy, the partition of CEO and executive, and monetary mastery of the chief decide authority style and significance (Fang et al., 2020). On the other hand, jobs and duties and the greatness of responsibility of authority influence the FP of an organization (Flanigan et al., 2017). Therefore, we formulate a hypothesis.

*H4: There is a mediating effect of HL on the relationship of CG and corporate FP*.

### MI as a Moderator

Incentives are utilized as an apparatus to inspire workers (Mata et al., 2021). They are usually either financial or non-financial. When leaders are lauded for extra rewards, they can be intrinsic or extrinsic remunerations in which they can be urged to take care of business. CG law requires that there is an arrangement of interests among executives and associations. It prompts a reduced amount of risk or misrepresentation and builds the inspiration for representatives to work in a manner important for the accomplishment of the association. Everybody is energized negatively or positively (Kanfer et al., 2017). Appropriate organizational leaders lead in the past utilizing different techniques to advance the impact on the association to expand their effectiveness and efficiency. It is important to disclose the thought process first to examine motivation (Guest, 2017).

For when individuals feel ownership of something, they feel constrained to take a gander at it. Organization leaders should impact their kin to make them more proficient and gainful. Powerful governance is exceptionally dependent on staff motivation. Organizational leaders should insist on the soul of the inspiration group so that representatives can work with a solid inspiration for a more prominent obligation to the association. In any case, much of the time, countless business supervisors do not comprehend the viable cycle of worker incentives. Also, they neglect to see that inspiration is a basic part of a corporate initiative. In the absence of powerful persuasive information, numerous corporate leaders cannot manage undesirable circumstances in associations. In the event that they had a working agreement, they would have the option to oversee and impact their kins effectively. Without incentives, the business will neglect to work viably and gainfully. Incentives and occupation satisfaction are connected (AlKahtani et al., 2021). Aguenza and Som (2018) noticed that the components that rouse workers are very similar things that diminish or obstruct work fulfillment. Powerful persuasive cycles and guaranteeing staff efficiency and request, tacticians consistently need to comprehend the elements that inspire representatives successfully. What is more, paying executives is perhaps the most promising exercise for a human asset the board. Organizational leaders should ensure the compensations of their workers to improve (Zhou et al., 2021). Jin and Fu (2019) clarified that the honors program contains approaches and the ways associations oversee representative compensations, for instance, by expanding yearly pay rates. This methodology effectively affects the enlistment and retaining of representatives. Work-based incentives persuade representatives and increment profitability and effectiveness. Individuals at work request wages and acknowledgment. The reward framework should be efficient and reasonable in this manner. On the off chance that chiefs neglect to do equity to the advantage of representatives, it will not propel them to build profitability; subsequently, they will not feel committed to play out their obligations. With regard to publicizing for a superior work, money is not generally the issue. Sometimes, organizational leaders can value somebody's work by sending an email or greeting to others out in the open, perceiving the great work of that individual, and impacting the worker to help their future exhibition (Zhou and Wu, 2018). These inquiries have resulted in motivational force programs that have a negative impact on the executive's position of influence. In this regard, we propose the following hypothesis:

*H5: MI as a moderating effect on the relationship of CG and HL*.

## Research Methodology

### Sampling Technique

This sample is the population configuration that depicts the entire population for this research, we utilized convenience sampling technique, which is limiting this impact of time and cost. According to Etikan et al. (2016), the sample size selection represents its target audience effectively as it is impossible for the researcher to get the sample from the whole population. Convenience sampling technique was used, and data were collected in 4 months, i.e., September, 2020 to January, 2021. Furthermore, based on the G^*^ power analysis, the minimum sample size required for this study should be 119 respondents to generate a power of 0.95 and a medium size effect of 0.15 (Faul et al., 2007). Researchers were able to obtain data from 300 target respondents, which was more than the minimal sample size required. As a result of the COVID-19 epidemic, data were also collected online *via* email and a Google survey form. Initially, 400 questionnaires were distributed to collect data from five cement manufacturing companies, namely, Bestway Cement Limited, Fauji Cement Company Limited, Askari Cement Limited, and Cherat Cement Company Limited located across Pakistan. Of 400 questionnaires, a total of 300 surveys were received and were usable, which were 75% of the total number of questionnaires distributed.

After data collection, Harman's single factor test is conducted to identify the common method variance, the result of extraction sums of squared loading is 25.44% of variance, which is less than 50%, meaning that there is no common method bias issue in the data (Tehseen et al., 2017). Of the 300 responses, most of the respondents were men (74.7%). Table 1 shows the gender formation of a sample in which 74.7% were male and 25.3% were female. The bulk of the people who responded were between the ages of 20 and 40, having the education of Bachelor, Masters, and MS, simultaneously. The percentage of respondents with project experience, which shows that the bulk of respondents have experience ranging from 6 to 10 years to more than 10 years. According to the data, respondents in the study have a wide range of employment experience. The data collected from the respondents who worked on projects as team members in the following roles. As we were to get the perspective of project managers, we can see that majority of them were senior managers and HR directors. Previous research illustrated that gender, age, education, and experience have a significant association with FP (i.e., Sar, 2018; Centobelli et al., 2019). The analysis of variance (ANOVA) results indicated that gender and position were found to be significant, while age, education, and experience are found to be non-significant, as shown in Table 1.

**Table 1:** Descriptive statistics.

**Demographics**	**Items**	**Frequency**	**Percent**	**Mean square**	**F**	**Sig**.
Gender	Female	76	25.3	0.421	2.340	0.007
	Male	224	74.7			
Age	20–30	106	35.3	0.495	0.862	0.587
	31–40	135	45.0			
	41–50	55	18.3			
	>50	4	1.3			
Education	Bachelor	77	25.7	0.789	1.291	0.223
	Masters	148	49.3			
	MS	64	21.3			
	PhD	11	3.7			
Experience	<1	22	7.3	0.902	1.229	0.262
	1–5	56	18.7			
	6–10	143	47.7			
	>10	79	26.3			
Position	Auditors/accountants	25	8.3	7.043	2.636	0.002
	Middle management	31	10.3			
	Senior managers (vice presidents)	86	28.7			
	Human resource directors	51	17.0			
	CEOs/presidents	43	14.3			
	Board of directors	40	13.3			
	Company shareholders	24	8.0			

## Results

### Measures

Five response possibilities on a Likert scale ranging from 1 to 5. “1 = strongly disagree” to “5 = strongly agree” were used to measure all items. The 25-items scale adopted from Mgammal et al. (2017) was used to analyze CG coving sub-domains (shareholders' right 4-items, regulatory framework 4-items, enforcement 4-items, ownership concentration 4-items, transparency, and disclosure 9-item scale). A 4-item scale developed by Centobelli et al. (2019) was used to measure FP. The mediator, a 9-item scale developed by Owens et al. (2013), was used to measure HL. Lastly, the moderator, a 5-item scale developed by Al-Belushi and Khan (2017), was put to calculate MI.

Anderson and Gerbing (1988) used a measuring model and a structural model to put our theory to the test. Covariance-based structural equation modeling (CB-SEM) in SPSS V.25 and AMOS V.22 was used to assess model fitness and hypotheses, as well as to construct the reliability and validity of the measurement model. Table 2 presents a breakdown of the variables and the resources from which they come, as indicated. The measurement technique indicated the validity and reliability of the constructs. A confirmatory factor analysis (CFA) based on the maximum likelihood technique was used to determine if the suggested models were valid (Brown, 2015). The estimation of internal consistency is executed with the help of a reliability coefficient, which is named as Cronbach's alpha and is denoted by the symbol (α) as the name shows alpha (Cronbach, 1951). The method measures the inter-correlation of items in a given questionnaire and establishes a scale. As shown in Table 2, all Cronbach's α above the threshold values are greater than 0.70 (Tavakol and Dennick, 2011). Data normality was checked for skewness and kurtosis. The value of each variable for skewness should vary between −1 and +1 and for kurtosis between −3 and +3. The normality analysis showed that all values were within the acceptable range, meaning that the data were normally distributed, as presented in Table 2 (Cain et al., 2017).

**Table 2:** Measurement model.

**Constructs**	**Factor loadings**	**α**	**Mean**	**S. D**	**Skewness**	**Kurtosis**	**Authors**
Corporate Governance		0.758	3.5317	0.52771	−0.125	−0.258	Mgammal et al. (2017)
*Shareholders' right*							
Minority shareholders' rights are protected	0.624						
All shareholders have the same rights to elect/remove members of the board	0.819						
Minority shareholders have the same rights to vote in general meetings	0.891						
Aggrieved shareholders have recourse	0.926						
Regulatory framework							
Rules and procedures for transactions are in place	0.907						
Commercial laws, stock market listing rules and regulations are in place	0.889						
Auditors are regulated	0.889						
Government agency responsible for enforcement of corporate laws	0.898						
*Enforcement*							
Inspectors investigate non-compliance with statutory requirements	0.825						
Inspectors investigate complaints by shareholders about mismanagement	0.714						
Inspectors investigate oppression of minority shareholders.	0.887						
Actions are taken against auditors' failure to report improper financial records	0.876						
*Ownership concentration*							
Have different compositions of ownership	0.668						
Minority shareholders are allowed to express their views at general meetings	0.694						
Chairpersons sometimes ignore minority shareholders at general meetings	0.529						
Preferential treatment is often given to large shareholders	0.676						
*Transparency and disclosure*							
Independent auditors	0.800						
Insider trading effectively prohibited	0.681						
Equal access to information for all shareholders	0.498						
Disclosure of managerial ownership and compensation	0.691						
Board of directors' responsibilities	0.721						
Reviewing and guiding corporate strategy	0.581						
Reviewing key executive and board compensation	0.420						
Ensuring the integrity of the corporation's financial reporting system	0.649						
Monitoring the effectiveness of the governance practices	0.645						
Humble Leadership		0.718	3.4911	0.65217	−0.263	−0.346	Owens et al. (2013)
This person actively seeks feedback, even if it is critical.	0.797						
This person admits it when they don't know how to do something.	0.687						
This person acknowledges when others have more knowledge and skills than him- or herself.	0.840						
This person takes notice of others' strengths.	0.770						
This person often compliments others on their strengths.	0.501						
This person shows appreciation for the unique contributions of others.	0.635						
This person is willing to learn from others.	0.882						
This person is open to the ideas of others.	0.776						
This person is open to the advice of others.	0.604						
Monetary Incentive		0.807	3.4467	0.81423	−0.269	−0.353	Al-Belushi and Khan (2017)
I give importance to monetary incentives	0.916						
Monetary incentives are of no value to me	0.752						
Monetary incentives offered matches my work effort	0.867						
Monetary incentives are not timely	0.824						
Monetary incentive is not up to my expectation level	0.760						
Financial Performance		0.856	3.4275	0.80157	−0.342	−0.369	Centobelli et al. (2019)
The return on investment of our company is higher compared to competitors.	0.803						
The return on assets of our company is higher compared to competitors.	0.568						
The sales growth and profitability of our company are higher compared to competitors	0.873						
The total operating costs of our company are lower compared to competitors	0.598						

*α, Cronbach alpha; SD, standard deviation*.

### Model Fit Analysis

We employed goodness-of-fit criteria to evaluate the theoretical framework in accordance with Byrne (2001). With values ≥ 0.90%, the structural modeling suggests that the proposed model is a good match. To accept or reject the tested model, CMIN/*df* , goodness-of-fit index (GFI), and adjusted fit index (AGFI), Bollen's incremental fit index (IFI), normed fit index (NFI), comparative fit index (CFI), and residual model error (RMSEA) were utilized (root mean square error of approximation). Following, we determined that CMIN/*df* values around 5.00 were suitable as CMIN was dependent on the sample size. When RMSEA was ≤ 0.08, GFI, NFI, and CFI values ≥0.95 were considered good fits in the literature. It is obvious that the proposed hypothetical model has a strong overall data fit when it comes to calculating CG, FP, HL, and MI. Table 3 shows that this structural model has a good fit (NFI = 0.910; IFI = 0.923; TLI = 0.956, CFI = 0.952, GF1 = 0.958, AGFI = 0.922, CMIN/df = 2.06, and RMSEA = 0.045).

**Table 3:** The analysis of model fit.

**Measurement models**	**NFI**	**IFI**	**TLI**	**CFI**	**GFI**	**AGFI**	**CMIN/df**	**RMSEA**
Threshold values	>0.9	>0.9	>0.9	>0.95	>0.95	>0.8	<3	0.05–0.1
Configural invariance (baseline model)	0.811	0.834	0.801	0.821	0.800	0.733	3.725	0.061
Metric invariance	0.910	0.923	0.956	0.952	0.958	0.922	2.06	0.045

### Correlation Analysis

The most useful tool and common type of analysis to test the relationship between two variables is correlation analysis. This analysis demonstrates a connection between two variables (indicated by the significance level). “A positive sign demonstrates that both the variables move similarly and a negative sign shows cases that variables have inverse developments” (Cohen et al., 2013). This also helps to remove the problem of multi-colinearity if it exists in the data. Correlation defines the linear relationship and the level of correlation between independent variables. The correlation coefficient for measuring the reliance between two variables is calculated using Pearson's correlation analysis. The value of coefficient lies between +1.00 and −1.00; however, a value of 0 indicates no correlation between the variables.

Correlation analysis between the study variables, i.e., gender, position, CG, FP, HL, and MI, has been demonstrated in Table 4. According to correlation (Table 4), the control variable gender was negatively correlated to FP (*r* = −0.143, *p* < 0.05) and CG (*r* = −0.055, *p* < 0.05) while it is significantly, positively correlated to HL (*r* = 0.024, *p* < 0.05) and ML (*r* = 0.026 *p* < 0.05). Also, position is significantly and negatively correlated to CG (*r* = −0.081, *p* < 0.05), FP (*r* = −0.133, *p* < 0.05), and ML (*r* = −0.058, *p* < 0.05) but positively correlated with HL (*r* = 0.04, *p* < 0.015). CG was positively and significantly correlated to HL (*r* = 0.507, *p* < 0.01), ML (*r* = 0.688, *p* < 0.01), and FP (*r* = 0.209, *p* < 0.01). HL is significantly and positively correlated to ML (*r* = 0.442, *p* < 0.01) as well as FP (*r* = 0.720, *p* < 0.01). Meanwhile, ML is significantly and negatively correlated to FP (*r* = −0.244, *p* = 0.01).

**Table 4:** Correlation analysis.

**Constructs**	**1**	**2**	**3**	**4**	**5**	**6**
1. Gender	1					
2. Position	0.091	1				
3. CG	−0.055	−0.081	1			
4. HL	0.024	0.048	0.507**	1		
5. ML	0.026	−0.058	0.688**	0.442**	1	
6. FP	−0.143*	−0.133*	0.209**	0.720**	−0.244**	1

*CG, Corporate governance; FP, financial performance; HL, humble leadership; MI, monetary incentive.*

* *p < 0.05,*

** *p < 0.01*.

### Mediation and Moderating Analysis

The methods of Hayes (2012) are utilized for mediation and moderation analysis. Model 4 is used for mediation analysis, whereas model 7 is used for moderation mediation analysis. Regression analysis is a technique that assesses whether two or more variables have a statistical link (association). Regression analysis demonstrates the degree to which an outcome variable depends on the predictor variable. It gives a comprehension of the way that how the estimation of the measurement variable changes when there is a variation in one or more independent variables. Therefore, it clarifies the causal connection between variables, while correlation analysis just explains the relationship between variables. The regression process is carried out using various tools (for example, Baron and Kenny, 1986) but here, for convenience and suitability of the study, Hayes (2017) process technique is utilized for investigation.

As indicated by Baron and Kenny (1986), Hayes, 2015 technique is obsolete in light of the fact that it forces a state of absolute impact of causation for intercession while in a few specialists' perspectives it is not essential and is even an obstruction in the method for checking genuine effect (Preacher et al., 2007; Preacher and Hayes, 2008). As per these analysts, an indirect effect through mediation is additionally conceivable regardless of whether no evidence of direct impact between predictor and result factors is found (Hayes, 2012). In addition, as information in sociologies is constantly dangerous due to the circumstances, nature and setting of the respondents, so the bootstrapping procedure for intercession in Hayes (2012) process technique builds the amiability of reasonable outcomes on the grounds that the example is separated into numerous little odds and ends and analysis is kept running on those littler measured subsamples.

The results in Table 5 demonstrated that CG has a substantial positive relation with FP (β = 0.5013, *p* < 0.01). The value of β shows the percentage change demonstrating that a 1 unit change in CG leads to a 0.5013 unit change in FP. The results indicate that almost 50.13% of the change is observed on FP, and the *p*-value of 0.000 indicates a higher level of significance, which provides a strong basis to accept H1. Furthermore, the direct effect of CG to HL (β = 0.4944, *p* < 0.001) and HL to FP (β = 0.8371, *p* < 0.001) are both positive and significant. Therefore, Hypotheses H2 and H3 are accepted.

**Table 5:** Mediation and moderation analysis.

**Hypothesis**	**Relationship between constructs**	**β**	**S.E**	**t** **Values**	**R^**2**^**	**ΔR^**2**^**	**p Values**	**LLCI**	**ULCI**
	Direct Effect								
	Gender	−0.2332*	0.0902	−2.5846			0.0102	−0.4108	−0.0556
	Position	−0.0385	0.0207	−1.8617			0.0636	−0.0792	0.0022
H1	CG->FP	0.5013**	0.1728	2.9018			0.0040	0.1613	0.8413
H2	CG->HL	0.4944***	0.0795	6.2163			0.0000	0.3379	0.6509
H3	HL->FP	0.8371***	0.1024	8.9092			0.0000	0.1388	0.7355
	Mediating Effect								
H4	CG->HL->FP	0.4944 X.8371 = 0.4138***	0.1590		0.7540	0.5686	0.0000	0.2759	0.6468
	MI->HL	0.2324**	0.0777	2.9904			0.0030	0.0795	0.3854
H5	Moderating effect	−0.6837***	0.0962	−7.1039	0.6362	0.4047	0.0000	−0.8731	−0.4943

*N = 300, Unstandardized regression coefficient reported. Bootstrap sample size was 5,000. CI = 95%.*

*CG, Corporate governance; FP, financial performance; HL, humble leadership; MI, monetary incentive; α, Cronbach alpha; SD, Standard deviation.*

* *p < 0.05,*

** *p < 0.01,*

*** *p < 0.001, LLCI, lower limit CI; ULCI, upper limit CI*.

**Figure 1:**
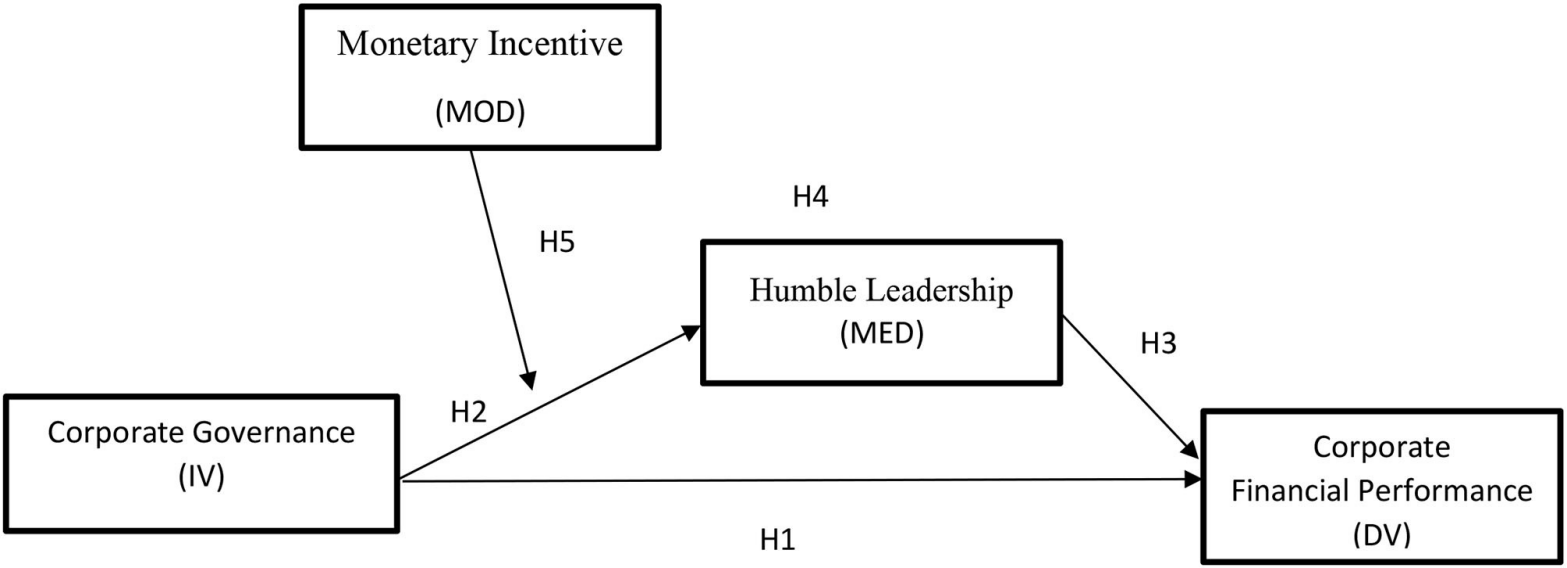
Conceptual model.

**Figure 2:**
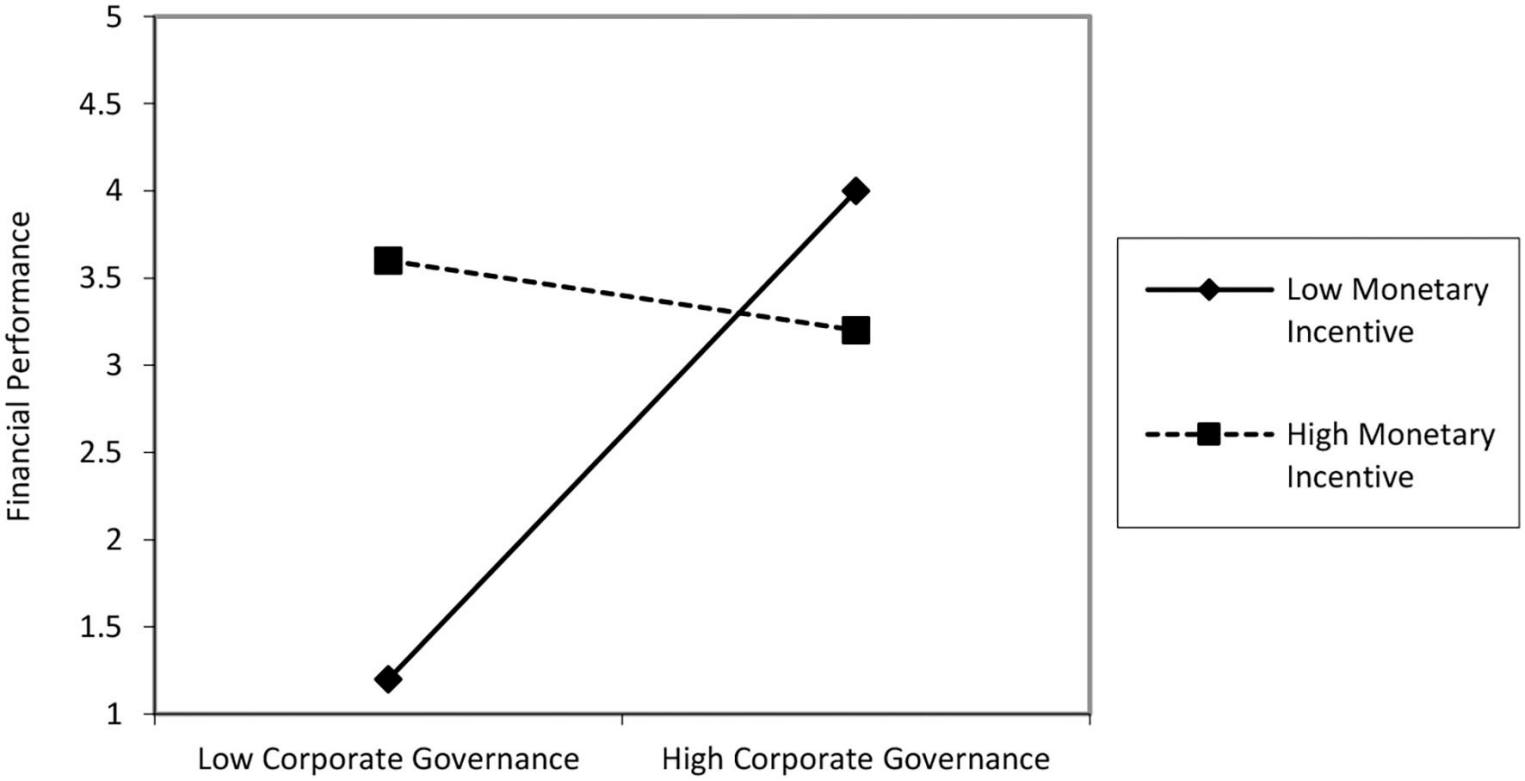
Interaction graph.

According to the results reflected in Table 5, it is evident that the mean indirect effect of CG on FP through the mediation of HL is positive and significant (β = 0.4138, *p* < 0.001). Bootstrapping values are lower limit CI (LLCI) = 0.2759 and upper limit CI (ULCI) 0.6468 with a 95% CI excluding 0. These results suggest sufficient support that HL completely mediates the relationship (see Figure 1). Hence, H4 is accepted. Lastly, H5 explained that MI moderates the relationship between CG and HL significantly but in a negative way such that CG × MI (β = −0.6837, *t* = −7.1039, *p* < 0.001). The finding shows that MI moderates negatively between CG and HL and the relationship is significant because the lower limit of value is −0.8731 and upper limit value is −0.4943. To check the moderating effect between MI, CG, and HL, the interaction graph in Figure 2 was calculated. The negative relation was proposed between CG and HL would be weaker in the presence of MI. The graph reflects the same results; if the MI is low, then the slope of graph is stronger. In other case, when MI is high, the relation between CG and HL becomes weaker and the slope line is steeper than the value of a moderator. Hence, H5 is rejected.

## Discussion

This research investigates the influence of CG on FP through the mediating role of HL and MI as a moderator on the relation between CG and HL in the context of cement manufacturing organizations in Pakistan. The result revealed that CG has a positive and significant effect on FP both directly and indirectly through the mediating variable HL. Governance standards express that the organization's targets should be met as per the association's standards and should be successfully executed, overlooked, moved, or changed to support individuals (Mohamad, 2018). Another significant factor in CG is the board setup. It implies how an association isolates its administrators and shows the significance of leadership roles in CG. Leadership has proven to be a decision in organizations and workers' actions in more than one sector. Leadership without significant risks of unreasonableness does not occur when active pioneers are seen to descend in some unacceptable way or making unique designs in a particularly inappropriate and ineffective method (Teece et al., 1997).

Leadership can build a dream that drives people together to work toward a common purpose of energy. Thus, this program is a loop in which one recruit others to fulfill a mission and ties them to such an entity to make it robust and understandable. The leadership finally created a path that the public would put forward to make something memorable possible. Apart from the direct causes of staff work and then the organization's success, two essential concerns are to consider the importance of leadership decisions on job satisfaction and organizational involvement (Haider and Tehseen, 2022). Motivational leaders can also allow their supporters to participate in decisions and encourage them to express their views (Hunter et al., 2007). Leadership has always been essential and more critical than ever in today's business world. Through research, efforts to improve the sector outreach can make a significant contribution (Sarwar, 2013). Consequently, by representing them and recognizing the importance of the task, leaders seem to inspire and encourage dependents. Revolutionary leaders too often have high expectations and values for themselves (Smith and Moore, 2019). They set expectations for the success of the organization, but they also involved themselves in the emergence of each of their assistants' skills. The success of an organization depends mainly on the people who work in the company. The leader is a critical member; therefore, the relationship between HL and corporate FP is positive (Zhou and Wu, 2018).

Furthermore, incentive programs aim to improve employee efficiency but can also contribute to increasing employee morale and morality. If workers see clear signs that their job is appreciated and valued as staff, they are more loyal to their employer. MIs allow employees to continue working toward their ambitions and helping the business meet its targets; therefore, MI has a moderating effect between CG and HL. Motivation is an effective strategy to motivate people to attain their objectives. Keeping staff is the company's most valuable asset. Employee retention is necessary in any firm. This guiding principle assists people to remain focused on the path to achievement in the face of adversity. When monetary factors (incentives) rise in the company, there are leaders and staff engage in negotiation and dispute just as a result of nepotism. Many leaders prefer their close relationships to work with them except others because they do not have good relationships with employees. Thus, the result revealed that MI negatively effects the relationship between CG and HL, hence H5 is rejected. To address this problem, organizations need to provide training, development opportunities, and reduce cultural differences between employees and leaders.

### Theoretical and Particle Implications

These findings have significant implications: Firstly, our research revealed new findings to bridge the gaps in the overall CG literature about the lack of consensus on the financial effects of CG procedures. The result adds to the knowledge by giving fresh and unique evidence that present CG systems are ineffective in mitigating the financial problem of manufacturing organizations in developing countries. Secondly, the positive influence of humble leaders on a corporate board may play key roles in increasing the FP of organizations. The humility of a team leader is crucial for team innovation, and businesses can encourage this characteristic during training, recruitment, and assessment. For example, leaders may be educated to demonstrate greater humility through performance evaluation and training programs leaders may be educated to demonstrate greater humility through performance evaluation and training programs (Haider et al., 2021). As crucial selection criteria, a company may also add characteristics such as admitting mistakes, complimenting subordinates' abilities, and learning from others. We also encourage leaders to develop their own competence and expertise to enhance the good impact of HL behavior. Moreover, while assessing the efficiency of a leader, a 360-degree review may be undertaken by the leader's superior, colleagues, and subordinates.

Thirdly, this research may assist senior-level decision makers in manufacturing organizations to enhance the most important variables and methods that can increase employee motivation in terms of MIs, hence achieving organizational goals and objectives. According to Aguenza and Som (2018), living things increase assumptions to understand about the consequences of behavior and behave in a manner that may alter the long-term outcomes. In general, the expectancy theory perspective recommends that the inspirational power an individual gets to choose a single activity from a larger set is another capacity of the apparent potential that such conduct will bring about the accomplishment of different valence impacts of these people's results. Expectancy theory emphasizes the importance of the phases of the organization of inspiration and how they relate and balance the effects of certain needs or preconditions that may influence conduct. According to the expectancy theory, the strength of a certain tendency to perform relies on the level of anticipation for a specific outcome and the need for the individual's performance. Moreover, the expectancy theory explained that an employee's motivation relies on his or her need for an incentive, the probability that the effort will deliver the desired outcomes (expectancy), and the belief that the incentive is a reward for hard work (instrumentality). Employee happiness after completing the objectives is only an anticipation, not a reality. Mata et al. (2021) have elucidated that the honors program includes the ways and manner in which organizations manage representational compensations, such as by increasing annual pay rates. Lastly, the ability of innovation of teams is enhanced when leaders encourage an environment conducive to ongoing learning (Khairuddin et al., 2021). Specifically, leaders may inspire subordinates to learn from others by serving as an exemplary role model. In addition, leaders may seek to impart their own successes to their subordinates through learning sessions. In addition, they may provide extra learning opportunities and establish learning incentives for team members. For instance, leaders may plan weekly learning and idea-sharing sessions to promote brainstorming and boost team innovation.

### Limitations and Future Directions

Despite the significant contributions of the current study, it has some limitations that provide considerable opportunity for future study. Firstly, only Pakistani manufacturing companies are included in this study scope and range. Future studies can test the current study model in other fields and environments (i.e., fashion industry and hotel industry). Secondly, the research is cross-sectional in nature as the environment of manufacturing companies is always changing, and the present study may not reflect future business situations. This restricts its generalizability; for future research, investigators should employ a longitudinal method. Lastly, future studies may improve the model by investigating additional mediators such as work satisfaction, top management support, employee empowerment, and knowledge sharing attitude. There are several different directions in which future researchers can go from here. The incorporation of important new factors may assist to raise the previously established grounds of study in this field.

## Data Availability Statement

The raw data supporting the conclusions of this article will be made available by the authors, without undue reservation.

## Ethics Statement

This research has been approved by the Research Ethics Committee of Xian University of Technology.

## Author Contributions

All authors listed have made a substantial, direct, and intellectual contribution to the work and approved it for publication.

## Funding

This research was done under the supervision of School of Economics and Management, Xian University of Technology, Xi'an, Shaanxi, China. This project is financed by the “Key projects of National Social Science Foundation of China in 2021 (21AJY020).

## Conflict of Interest

The authors declare that the research was conducted in the absence of any commercial or financial relationships that could be construed as a potential conflict of interest.

## Publisher's Note

All claims expressed in this article are solely those of the authors and do not necessarily represent those of their affiliated organizations, or those of the publisher, the editors and the reviewers. Any product that may be evaluated in this article, or claim that may be made by its manufacturer, is not guaranteed or endorsed by the publisher.
